# Quality of life and pain severity changes over time in patients with breast cancer who were referred for palliative oncology treatment in Indonesia: a hospital-based cohort study

**DOI:** 10.3389/fgwh.2025.1537824

**Published:** 2025-10-14

**Authors:** Dwi Gayatri, Ljupcho Efremov, Andreas Wienke, Rafael Mikolajczyk, Eva J. Kantelhardt

**Affiliations:** 1 Institute for Medical Epidemiology, Biometrics, and Informatics (IMEBI), Interdisciplinary Center for Health Sciences, Medical Faculty of the Martin Luther University Halle-Wittenberg, Halle (Saale), Germany; 2 Department of Epidemiology, Faculty of Public Health, Universitas Indonesia, Depok, Indonesia; 3 Department of Radiation Oncology, University Hospital Halle, Halle (Saale), Germany; 4 Department of Gynecology, University Hospital Halle, Halle (Saale), Germany

**Keywords:** quality of life, advanced breast cancer, pain severity, EORTC QLQ-C15-PAL, Indonesia

## Abstract

**Introduction:**

Understanding quality of life (QOL) and changes in pain severity over time is important to quantify cancer patients' treatment outcomes. However, this information is limited, especially in lower-middle-income countries. This study aimed to prospectively assess QOL, QOL domains, and pain severity over time in patients with advanced breast cancer in Indonesia.

**Methods:**

Women with advanced breast cancer (*n* = 160) who were referred to the palliative oncology unit were enrolled in the study. They completed the European Organization for Research and Treatment of Cancer QOL Questionnaire for advanced cancer patients (EORTC QLQ-C15-PAL) and the visual analog scale (VAS) for pain severity at three assessment points, namely, at baseline (T0) and at 3- (T1) and 6-month (T2) follow-ups. The repeated-measures analysis of variance model was used to assess changes over time after adjusting for age, place of residence, marital status, and Karnofsky Performance Status score. Change over time was classified into three groups, namely, deterioration, improvement, or a small difference.

**Results:**

The descriptive analysis showed that the patients’ EORTC QLQ-C15-PAL mean scores for overall QOL and the functional scales (physical and emotional functioning) were fair to good at all assessment points (range: 60–70 points) and substantially better at T0 compared to T1 and T2. In addition, most of the scores for the symptom scales of the EORTC QLQ-C15-PAL indicated lessening symptom burden (<10 points), except for pain and fatigue (20–30 points). At the same time, overall QOL, emotional functioning, fatigue, dyspnea, appetite loss, constipation, and VAS score remained stable over time. Exceptions were found for physical functioning (a medium to large deterioration with scores of −16.5 and −19.8 points, respectively) and insomnia (a medium improvement with a score of −13.4 points), with clinically relevant changes.

**Conclusions:**

Our findings from a 6-month longitudinal study show that palliative oncology treatment benefitted patients with advanced breast cancer in this health facility across several symptom scales.

## Introduction

Breast cancer is a leading cause of mortality in women worldwide and especially in low- and middle-income countries (LMICs), where most cases are diagnosed at an advanced stage (1). Patients with advanced cancer often experience multiple symptoms and functional deficits (2, 3). Palliative care (PC) can be employed for symptom control along the disease trajectory and increases the quality of life (QOL) in this group of patients (2). QOL in the cancer context is an important multidimensional patient-reported outcome, encompassing physical, emotional, social, and cognitive functions related to patients' perceptions and expectations of their health status and symptoms (4).

Symptoms, i.e., anxiety, depression, pain, fatigue, dyspnea, and appetite loss, often negatively affect QOL and the cancer patient's ability to perform daily activities (5, 6). Cancer-related symptoms can occur due to disease progression or as a side effect of cancer treatment. For example, untreated chronic pain in cancer patients often also worsens QOL in other domains, i.e., fatigue, nausea, constipation, sleep disturbances, and depression (7, 8). Patients with advanced cancer experience multiple symptoms during their illness trajectory that can often fluctuate in intensity, which means that repeated QOL measures are needed to identify and control these cancer-related symptoms to improve their QOL, especially in advanced cases who have limited options for further treatment (9, 10). Moreover, assessing changes in QOL, QOL domains, and pain severity in patients with advanced cancer is necessary to detect their improvement, stability, or deterioration with PC treatments over time (11). Furthermore, advanced interventions, e.g., using artificial intelligence, should be considered as a potential strategy in this research context (12), which would allow healthcare providers to optimize cancer management strategies to improve or maintain patients' QOL. Unfortunately, in many LMICs, including Indonesia, QOL is not routinely assessed, and most QOL studies in this context were cross-sectional or prospective studies with limited follow-up (13, 14). Therefore, this study aims to prospectively assess the change in QOL score, QOL domains' scores, and pain severity in patients with advanced breast cancer who were referred for palliative oncological treatment in Indonesia.

## Patients and methods

The Strengthening the Reporting of Observational Studies in Epidemiology guideline was followed in this study (15).

### Study population

Patients with advanced breast cancer (*n* = 160) who were referred to the palliative oncology unit at the oncology department of the “Dharmais” Cancer Hospital in Jakarta, Indonesia, between January and February 2020, were invited to participate in this study. The palliative oncology treatment in this health facility has non-curative intent and could include chemotherapy or hormonal therapy, while radiotherapy and surgery were usually reserved for when no other means to reduce symptoms were available (16). Our research was an exploratory study with a convenience sampling approach, as no previous prospective data exist on this research context in Indonesia. For a single exploratory group and three measurements to detect a medium-sized effect (0.20) with a study power of 0.80 and alpha of 0.05, the study required 83 participants (17). Considering a dropout rate of 10%, the total sample size needed in this study was 91. Patients who agreed to participate were followed up over a 6-month observation period, ending in September 2020. The inclusion criteria were breast cancer stage III or IV (18), age >18 years, and being referred for palliative oncological treatment for the first time during the study enrollment period (baseline). The criteria for exclusion were the presence of psychological disorders (self-reported and verified by one’s medical record during the study enrollment period by a nurse) and the inability to respond to the questions independently.

### Data collection procedures

Potential respondents who met the inclusion criteria were screened by a nurse during the registration process at the oncology unit before their consultation session with the oncologist. Respondents were recruited using convenience sampling. Eligible patients who met our recruitment criteria received a detailed explanation of the study's objectives, procedures, and potential risks. Written informed consent was obtained from all the respondents prior to completing the data collection. The respondents were informed of their right to refuse participation or withdraw at any time without penalty in this study. Copies of the study instrument were given to all respondents as a reference for future follow-ups. In addition, the study instrument was sent in a digital format to each participant 1 day before each scheduled follow-up. The participants completed the study questionnaires at three timepoints: T0 (baseline), T1 (3-month follow-up), and T2 (6-month follow-up). It is suggested that for patients with advanced cancer, a QOL assessment at baseline should represent the first step, while the follow-up examinations can best describe the clinical progression of patients with cancer during the PC phase (19). Therefore, each patient was contacted by telephone and requested to complete the study questionnaire at each scheduled assessment timepoint, irrespective of whether they completed their study questionnaire at the previous assessment time. The time and place of the next follow-up assessment were arranged at the patient's convenience as stated in the informed consent. The study was approved by the “Dharmais” Cancer Hospital Ethics Committee (136/KEPK/VII/2019) and by the Medical Faculty Ethics Committee at Martin Luther University of Halle-Wittenberg (2021-139), and followed the recommendations of the Declaration of Helsinki (20).

### Outcome measures

The study's primary outcome is the overall QOL score, but the QOL domains and pain severity score [visual analog scale (VAS)] were also assessed as additional outcomes. All the patients completed the Indonesian version of the European Organization of Research and Treatment for Cancer Quality of Life Questionnaire for Advanced Cancer (EORTC QLQ-C15-PAL), with a Cronbach's alpha coefficient between 0.70 and 0.85 (21), and the VAS at T0, T1, and T2. The EORTC QLQ-C15-PAL includes a total of 15 items and comprises functional scales (two items), symptom scales (seven items), and overall QOL or global health status (one item). A higher score (toward 100 points) on the overall QOL and functional scales describes a better overall QOL and functioning, while for the symptoms scale, scores toward 100 points indicate a worse state with more severe symptoms. All the EORTC QLQ-C15-PAL items were related to how the patient was feeling during the previous week before the assessment at T0, T1, and T2, with a 1-week window before and after the fixed date of assessments to increase the probability of response. The VAS was used to assess the patients’ pain severity status, ranging from 0 (indicating no pain) to 10 (representing the worst possible pain). The VAS is recognized for its reliability and validity in pain assessment (22). Pain severity, as measured by the VAS at three distinct time points, reflects the patients' current pain intensity at each assessment.

### Clinical and sociodemographic variables

A custom questionnaire was used to collect the participants’ sociodemographic characteristics (age, place of residence, education, marital status, religion, and ethnic group) and clinical characteristics (body mass index, Karnofsky Performance Status (KPS), metastasis status, and history of cancer treatments) at baseline. The KPS describes patients' functional capacity in daily activities on a scale from 0 (representing death) to 100 (representing normal activity) (23).

### Statistical analysis

Descriptive statistics, i.e., mean values with standard deviations (SD) and absolute and relative frequencies, were used to summarize the sample characteristics, all the EORTC QLQ-C15-PAL items, and pain severity (VAS score). The scoring of the EORTC QLQ-C15-PAL domains was performed according to the EORTC QLQ-C30 Scoring Manual (24) and its addendum (25). The EORTC QLQ-C15-PAL scoring principle was used to calculate the mean values for all the items (the raw score), which were then linearly transformed to yield scores from 0 to 100.

The normality of the data and model residuals was assessed using histograms and the Shapiro–Wilk test. When the assumption of homogeneity of variance and the covariance matrix was violated, the within-subjects effect values were corrected using the Greenhouse–Geisser or Huynh–Feldt method. The *p*-values were used for explanatory purposes only and were not adjusted for multiple comparisons (26). The repeated-measures ANOVA was used for the analysis and included a within-subjects factor (time) and scores at three timepoints: T0, T1, and T2. The model was adjusted for age, place of residence, marital status, and KPS score at baseline (27). This model was used separately for QOL, each QOL domain, and VAS. For missing values for any of the EORTC QLQ-C15-PAL items during the study, we used the multiple imputation approach with regression models to mitigate an uneven number of respondents at each timepoint. The multiple imputation approach was performed using five imputations for each follow-up before merging them into one complete dataset for the analysis (24). We included participants who were missing at later follow-up timepoints. To assess representativeness, we compared the participants’ sociodemographic characteristics between the timepoints. The partial eta squared ($\eta_{p}^{2}$) method was used to measure the effect size (small: 0.01; moderate: 0.06; and large: 0.14).

As a statistical association can sometimes be achieved for small changes in patient-reported outcome measures that might not be clinically meaningful, minimal important differences (MIDs) were additionally used as a guideline for interpreting mean change over time that is clinically relevant (28). The MID is defined as the “smallest difference in score in the domain of interest that patients perceive as important, either beneficial or harmful, and which would lead the clinician to consider a change in the patient's management” (29). To illustrate the clinically relevant differences in the variation in outcomes, the within-subjects changes in EORTC QLQ-C15-PAL and VAS scores were categorized into three classes, i.e., deterioration, small difference, or improvement. The classes were distinctive for each domain and were defined by a MID of 5 points, explaining a small, medium, or large change (28). Clinical relevance was considered if there was a 10-point difference (30). All the statistical analyses were performed using IBM SPSS Statistics version 25.0.

## Results

### Patient characteristics

**Figure 1:**
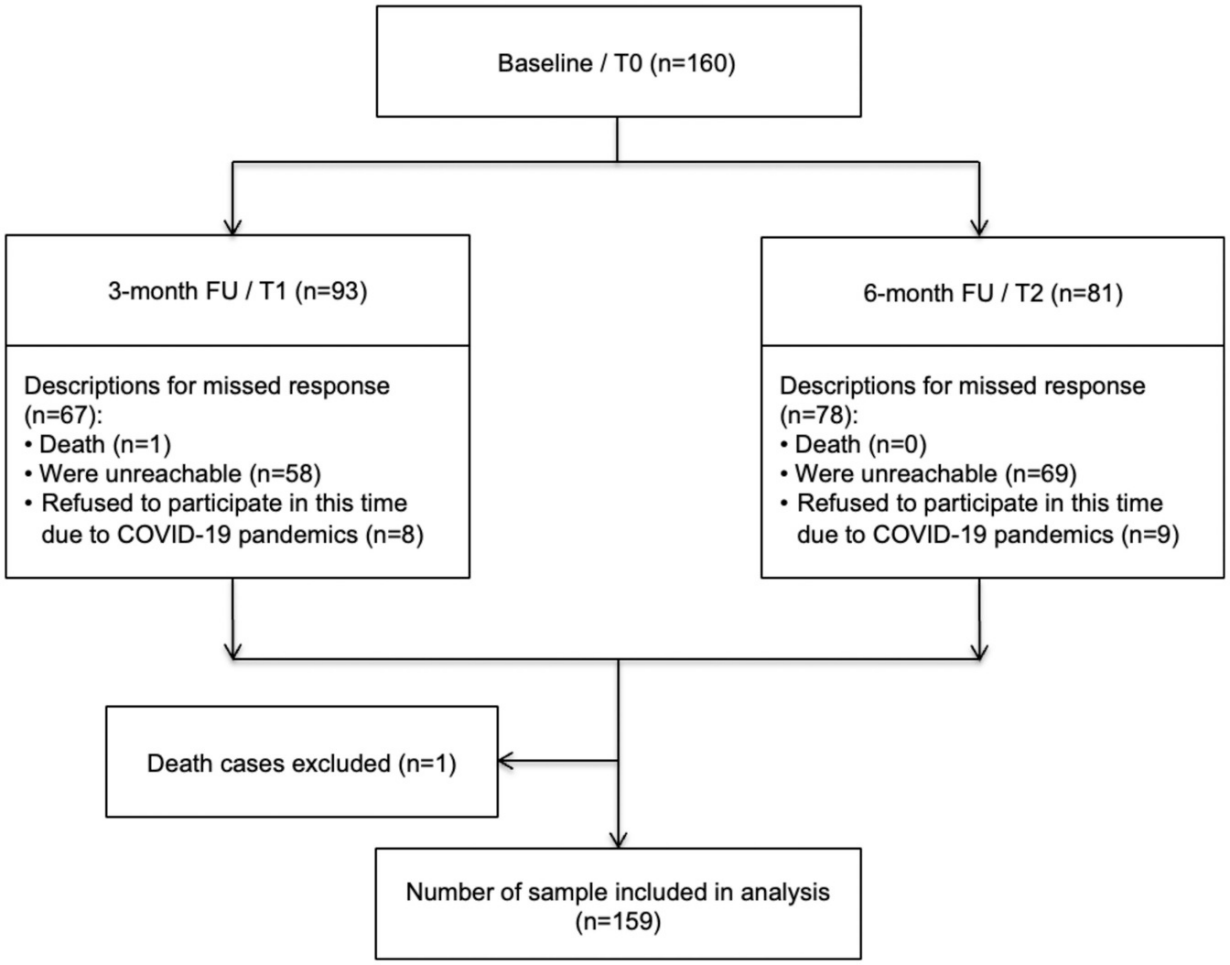
A flowchart of the study.

Of the 160 patients who completed the questionnaire at the baseline assessment, one patient died between T0 and T1 and was excluded from the analysis (Figure 1). Moreover, 93 (58.5%) and 81 (50.9%) respondents were willing to fill out the questionnaires (respondent retention) at T1 and T2, respectively. The common reasons for non-response at both follow-ups were being unreachable, with 36% at T1 and 43% at T2, and issues related to the COVID-19 pandemic, with 5% at T1 and 6% at T2 (Figure 1). The mean age of the patients was 50 ± 8.3 years at baseline. The majority of the patients lived in urban areas (72.3%), had a high school education (71.7%), were married (81.8%), and had a good mean KPS score (>65 points) (Table 1).

**Table 1:** Sociodemographic characteristics of the patients with advanced breast cancer at baseline (*n* = 159).

Characteristics	*N* (%)
Age (years)^a^; min–max	50.2 ± 8.3; 29–76
Karnofsky Performance Status (%)^a^	80.7 ± 6.9
Body mass index (kg/m^2^)^a^	25.8 ± 4.7
Systolic blood pressure (mmHg)^a^	128.7 ± 16.6
Diastolic blood pressure (mmHg)^a^	80.6 ± 9.9
Place of residence	
Rural	44 (27.7)
Urban	115 (72.3)
Educational level	
Never/primary/junior/senior high school	114 (71.7)
Vocational/under/postgraduate degree	45 (28.3)
Marital status	
Single/separated/widow/widower	29 (18.2)
Married	130 (81.8)
Religion	
Islam	137 (86.2)
Protestant	14 (8.8)
Catholic	7 (4.4)
Buddhist	1 (0.6)
History of surgery	
Yes	154 (96.9)
No	5 (3.1)
Did not know	0 (0.0)
History of radiation	
Yes	59 (37.1)
No	97 (61.6)
Did not know	3 (1.9)
History of chemotherapy	
Yes	100 (62.9)
No	55 (34.6)
Did not know	4 (2.5)
Metastasis status	
Yes	32 (18.2)
No/did not know	127 (79.4)
Pain therapy	
Yes	8 (5.0)
No	151 (95.0)

a Mean ± standard deviation.

### Quality of life score

**Figure 2:**
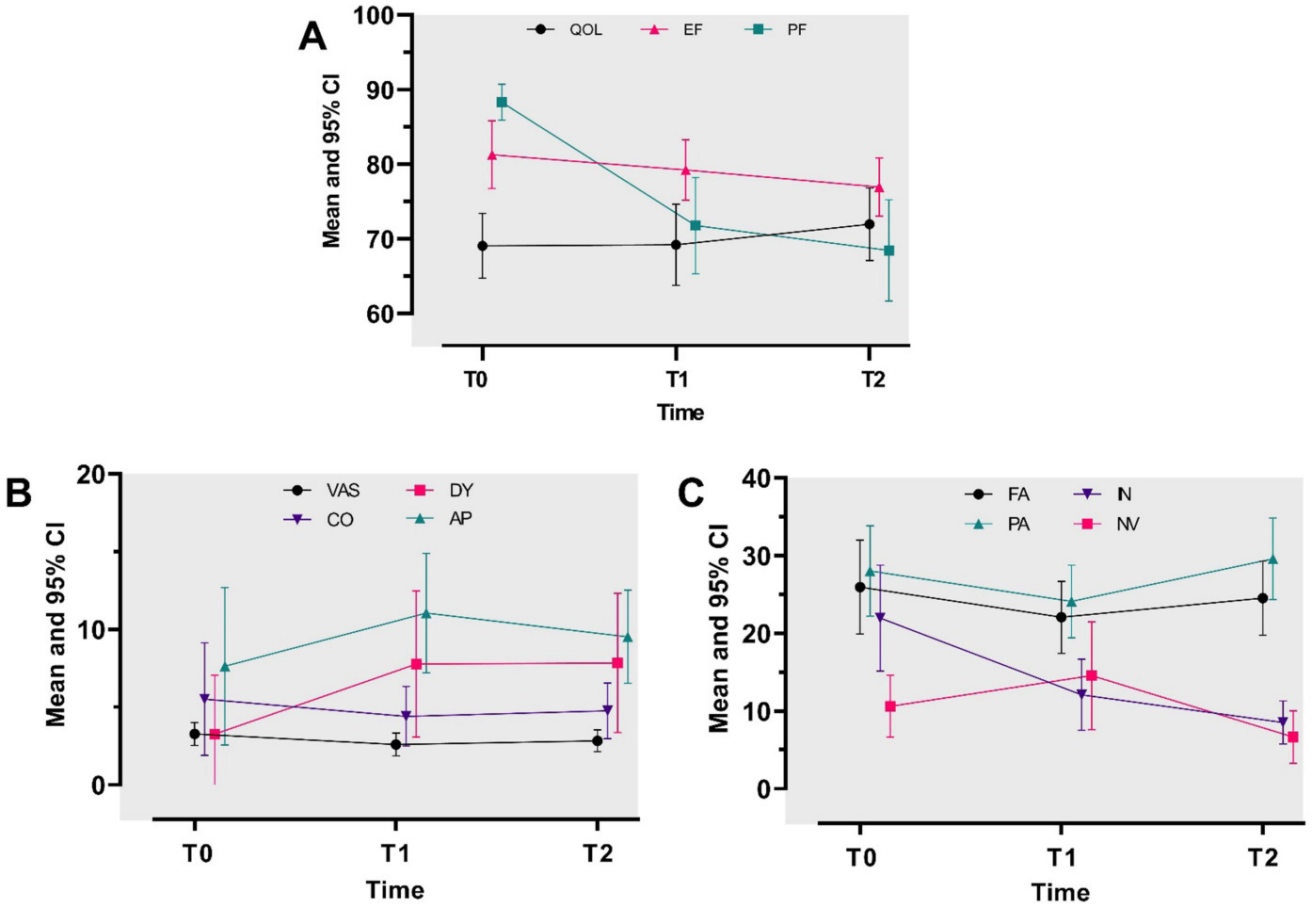
Repeated-measures analysis of variance for study outcomes at baseline (T0) and at 3-month (T1) and 6-month (T2) follow-ups. (**A**) Mean adjusted scores for QOL, EF, and PF. A mean score toward 100 describes a good QOL/EF/PF. (**B**) Mean adjusted scores for pain severity (VAS), constipation (CO), dyspnea (DY), and appetite loss (AP). (**C**) Mean adjusted scores for fatigue (FA), pain (PA), insomnia (IN), and nausea/vomiting (NV). A mean score toward 100 describes a high level of VAS/CO/DY/AP/FA/PA/IN/NV (**B**,**C**).

The EORTC QLQ-C15-PAL scores for overall QOL at all three assessments were stable, with fair scores (score range: 60–70) (Figure 2A; Supplementary Tables S1, S2). In the repeated ANOVA model, no association was found between overall QOL scores and time. Furthermore, the score differences over the examined timepoints were less than 10 points, showing no MID (Table 2).

**Table 2:** Repeated-measures analysis of variance for mean adjusted differences in quality of life, functional scales, symptom scales, and pain severity at baseline and at the 3- and 6-month follow-ups.

Variable	T0–T1			T0–T2			T1–T2		
	Difference in mean score (95% CI)	$\eta_{p}^{2}$	*p*-value	Difference in mean score (95% CI)	$\eta_{p}^{2}$	*p*-value	Difference in mean score (95% CI)	$\eta_{p}^{2}$	*p*-value
Global quality of life^a^	0.1 (−7.1 to 7.4)	0.006	0.97	2.9 (−3.9 to 9.7)	0.016	0.18	2.8 (−1.3 to 6.8)	0.006	0.18
Functional scales^a^									
Physical functioning	−16.5 (−23.4 to −9.7)	0.012	<0.001^c^	−19.8 (−27.2 to −12.5)	0.019	<0.001^c^	−3.3 (−7.5 to 0.8)	0.004	0.11
Emotional functioning	−2.0 (−7.8 to 3.7)	0.003	0.48	−4.3 (−10.3 to 1.7)	0.013	0.16	−2.3 (−5.5 to 0.9)	0.012	0.17
Symptom scales^b^									
Fatigue	−3.9 (−11.0 to −3.3)	0.007	0.29	−1.4 (−8.9 to 6.2)	0.002	0.71	2.4 (−0.8 to 5.7)	0.014	0.14
Nausea and vomiting	3.9 (−3.5 to 11.4)	0.007	0.30	−4.0 (−8.9 to 1.0)	0.017	0.12	−7.9 (−13.1 to −2.7)	0.058	0.003^c^
Pain	−3.9 (−10.2 to 2.4)	0.008	0.22	1.6 (−5.7 to 8.8)	0.002	0.67	5.5 (1.2 to 9.7)	0.003	0.012^c^
Dyspnea	4.5 (−0.5 to 9.4)	0.070	0.07	4.6 (−0.6 to 9.8)	0.049	0.08	−0.7 (−3.4 to 3.5)	0.021	0.96
Insomnia	−9.8 (−17.1 to −2.6)	0.045	0.008^c^	−13.4 (−19.9 to −6.9)	0.098	< 0.001^c^	−3.6 (−6.9 to −0.2)	0.028	0.04^c^
Appetite loss	3.4 (−2.2 to −9.1)	0.009	0.23	1.9 (−3.6 to 7.4)	0.011	0.49	−1.5 (−4.7 to 1.7)	0.012	0.35
Constipation	−1.1 (−5.0 to 2.8)	0.002	0.57	−0.7 (−4.6 to 3.1)	0.001	0.70	−2.6 (−5.1 to −0.2)	0.004	0.03^c^
Visual analog scale	−0.7 (−1.6 to 0.3)	0.012	0.18	−0.4 (−1.4 to 0.5)	0.006	0.37	0.4 (−1.4 to 2.1)	0.005	0.68

T0, baseline; T1, 3-month follow-up; T2, 6-month follow-up; CI, confidence interval; $\eta_{p}^{2}$, partial eta squared.

a Minus sign describes a deterioration trend for quality of life/physical or emotional functioning.

b Minus sign describes an improvement trend for symptom or pain severity (visual analog scale).

c A *p*-value < 0.05 was considered significant. The model was adjusted for age, place of residence, marital status, and Karnofsky Performance Status score at baseline.

### Quality of life domains and pain severity scores

#### Functional scales

The EORTC QLQ-C15-PAL scores for physical and emotional functioning (EF) at all three assessments decreased over time, with a high score (>70 points) (Figure 2A; Supplementary Table S2). The repeated ANOVA model demonstrated that the mean physical functioning (PF) score showed a medium (score differences ranging from −17 to −10 points) to large (score differences larger than −17 points) deterioration at the follow-ups compared to baseline (Table 2). Furthermore, the physical functioning score demonstrated a MID. However, the emotional functioning scores showed small differences among the three assessments, with no MID (Table 2).

#### Symptom scales and pain severity (VAS)

The EORTC QLQ-C15-PAL scores for most QOL domains (except for fatigue and pain) and VAS were descriptively low (less than 20 points) at all three assessments (Figure 2A; Supplementary Table S2). In addition, our model showed there was a small difference in the mean score (<10 points: no MID) across the three timepoints for most QOL symptom scale domains, i.e., fatigue, pain, dyspnea, appetite loss, constipation, and VAS. The mean nausea and vomiting score demonstrated a small improvement, starting at T1, but showed no MID (less than 10 points) (Table 2). A medium to small improvement was found for insomnia, beginning at T0, and showed a MID (larger than 10 points) (Table 2).

## Discussion

This study prospectively assessed overall QOL, QOL domains, and pain severity scores among Indonesian patients with advanced breast cancer who were receiving palliative oncology treatment. Our findings showed that the general QOL score was stable over the 6-month study period. Similarly, the scores for the majority of the QOL domains and pain severity demonstrated no statistically different changes and no changes in clinical relevance, while, interestingly, an improvement was identified for insomnia. Our study, with an overall QOL score of 68.1, was in line with findings from Brazil that showed that palliative care exposure positively affects the QOL of patients with advanced cancer, reporting an overall QOL score of 66.7 (31, 32). Clinicians should interpret QOL assessment scores by associating them with the magnitude of the change observed (effect size) and its practical implications, e.g., understanding the potential real-world impact of the observed change (33).

### Quality of life among patients with breast cancer

Patients with breast cancer follow disease trajectories of clinical stability for a long period, often maintaining comfort and relatively normal functioning, and then experience a rapid decline in their final weeks before death (3). Our results supported this finding; however, our study showed a much more stable trend during the 6-month follow-up period, probably due to this being a shorter follow-up period compared to the previous studies (10, 34). The main explanation is that experiencing long-term disease treatment provides the patients with sufficient time to accept and adapt to their situation (35, 36). For example, the optimal stress response to long-term disease is associated with living and working environments, resulting in the patients having the opportunity to control their environment, e.g., adequate coping resources (35). In addition, the psychobiological aspects emphasize that both resistance and vulnerability to stress are influenced by factors, e.g., how individuals cope with stress, their personality traits, and their social support they receive (35, 37). These factors play an important role in helping patients adapt to or manage the stress associate with chronic illness (35, 37). Furthermore, studies have emphasized that QOL in patients with advanced cancer varies due to different demographic and cultural characteristics (13). It is evident that cultural aspects play a key role, as family support and religion can help patients with advanced breast cancer accept their health situation (13, 37). Consequently, adaptation leads to resilience, which can maintain advanced cancer patients' QOL/QOL domains (37). Moreover, the PC approach to alleviating suffering and enhancing comfort may prevent further deterioration of QOL/QOL domains and stabilize symptom progression over time. For instance, the use of artificial intelligence in PC as an advanced technological intervention could be an effective strategy for improving cancer patients' QOL (12). Another possible explanation is the lack of information from patients who refused to participate in this study at study enrollment due to their weak physical condition, which may have contributed to a non-response bias (38). Patients with advanced cancer experience multiple symptoms during their disease trajectories that can fluctuate in intensity. Therefore, it is necessary to conduct a QOL assessment as a screening process to identify and treat symptoms early and on a continuous basis in this patient group.

### Quality of life domains (functional and symptom scales) and pain severity in patients with breast cancer

Patients with breast cancer commonly report multiple cancer-related symptoms, such as pain and fatigue, during cancer treatment. However, the majority of the QOL domains (physical and emotional functioning) in our study did not show changes over time during the palliative oncology treatment. This can be explained by missing patients in poor condition at baseline due to the convenience sampling method used and the short study observation time. In addition, patients with advanced cancer not only experience different levels of QOL but their QOL also changes in different ways in their disease trajectory (9, 11). Therefore, there is a need for regular assessment of the patient’s subjective QOL to provide care that extends life while maintaining the patient's QOL (11). This can be achieved through routine QOL screening to identify patients’ symptoms (39) and changes in their QOL/QOL domain score, leading to the development of an optimal treatment plan that will benefit the patients and meet their preferences (40). This is especially important in the treatment of patients with advanced cancer, as the decision for therapy is not easily reached.

Patients with breast cancer often experience a high cancer-related symptom burden. We observed no significant changes in symptoms during the studied palliative treatment, but surprisingly, an improvement in insomnia was reported. Though not often, this effect has been reported by other studies conducted in LMICs. For example, longitudinal studies compared patients with breast cancer who received a PC consultation and patients who received standard care without a PC consultation. Those who received a PC consultation had better QOL, emotional and social functioning, and less insomnia and depression (8, 41). A potential explanation could be related to the breast cancer clinical pathway, as breast cancer treatment with a curative intent, i.e., chemotherapy, negatively affects patients' sleep quality (42, 43). Studies have found that 30% of patients with breast cancer develop insomnia as a new problem and 25% reported a worsening of chronic insomnia (42, 43). Similarly, the dose-dense and custom treatment strategy is prominent in cancer treatment that negatively affects patients' QOL, but once the cancer treatment ends, the QOL/QOL domains improve (43, 44). Patients with breast cancer who are referred to PC no longer receive intensive curative treatment. Consequently, the main causes of insomnia may suddenly decrease, leading to other QOL domains, i.e., functional scales (physical and emotional functioning) and depression/anxiety improvement. Identifying and addressing insomnia in patients with cancer is crucial for clinicians and breast cancer programs because of its immediate impact on distress and QOL (45). Moreover, insomnia may contribute to subsequent adverse outcomes, affecting both physical and emotional health (46). Therefore, addressing insomnia in this patient group is necessary to maintain or improve overall QOL and other QOL domains.

### Strengths and limitations

This study provided additional information on QOL in patients with advanced breast cancer during their PC from a longitudinal perspective in Indonesia. However, several limitations existed. For instance, we used a convenience sampling approach and only observed one group of patients in one hospital, resulting in a reduction in statistical power and limiting the generalizability of our findings to a broader population. The self-reported approach has limitations, including the potential for reporting bias related to metastatic disease status, as patients may occasionally omit unfavorable information. Moreover, information about the exact cancer treatment received, e.g., adverse events/complications, was not possible to obtain in detail due to the nature of the study. However, since hospital nursing staff assisted in patient screening according to the eligibility criteria, we considered the medical information to be reasonably reliable. In addition, several cultural aspects, e.g., social support and religion/beliefs, that may explain this study's findings were not assessed. Six months is a considerable period of time for follow-up; however, Hinz et al. suggest that longer time periods are necessary to study changes in QOL in this research context (47).

## Conclusions

This study found that overall QOL, the majority of the QOL domains except insomnia, and pain severity scores were stable over 6 months in a cohort of patients with advanced cancer who received PC in an Indonesian setting. Assessing these domains using a longitudinal approach is important for capturing patients' cancer-related symptoms.

## Data Availability

The raw data supporting the conclusions of this article will be made available by the authors, without undue reservation.
