# Supplementary material for: Quality of life and pain severity changes over time in patients with breast cancer who were referred for palliative oncology treatment in Indonesia: a hospital-based cohort study
Source: Front Glob Womens Health. 2025 Oct 14;6:1537824. doi: 10.3389/fgwh.2025.1537824 (PMC12558998; doi:10.3389/fgwh.2025.1537824)
Supplement: Supplementary file 1 [file Table1.docx]

Supplementary Material

# Supplementary Tables

**Supplementary Table 1.** Repeated measures analysis of variance for mean unadjusted quality of life, functional scales, symptom scales, and pain severity during baseline, three- and six-month follow-up.

| **Variable** | **T0** | **T1** | **T2** |
| --- | --- | --- | --- |
|  | **Mean ± SD** | **Mean ± SD** | **Mean ± SD** |
| **Global quality of life** (15) | 68.09 ± 15.36 | 67.30 ± 17.46 | 70.96 ± 15.43 |
| **Functional scales** |  |  |  |
| Physical functioning (1-3) | 89.87 ± 12.36 | 64.56 ± 29.49 | 61.92 ± 30.71 |
| Emotional functioning (13, 14) | 81.49 ± 20.59 | 74.95 ± 18.76 | 72.64 ± 18.15 |
| **Symptom scales** |  |  |  |
| Fatigue (7, 11) | 22.49 ± 28.29 | 27.17 ± 21.46 | 29.19 ± 21.60 |
| Nausea and vomiting (9) | 4.30 ± 15.26 | 16.36 ± 22.14 | 9.03 ± 10.58 |
| Pain (5, 12) | 22.96 ± 27.55 | 28.63 ± 21.41 | 34.23 ± 23.66 |
| Dyspnea (4) | 3.25 ± 11.78 | 10.49 ± 14.95 | 9.45 ± 14.35 |
| Insomnia (6) | 17.61 ± 33.19 | 16.78 ± 20.49 | 10.49 ± 12.24 |
| Appetite loss (8) | 8.59 ± 24.00 | 12.59 ± 17.16 | 10.71 ± 13.43 |
| Constipation (10) | 4.19 ± 17.18 | 4.94 ± 8.92 | 5.88 ± 8.00 |
| **Visual analogue scale** | 2.1 ± 2.5 | 2.8 ± 2.4 | 3.2 ± 2.3 |

*EORTC QLQ-C15-PAL* European Organization for Research and Treatment of Cancer, *T0* baseline, *T1* three-month follow-up, *T2* six-month follow-up, *SD* standard deviation

**Supplementary Table 2.** Repeated measures analysis of variance for mean adjusted quality of life, functional scales, symptom scales, and pain severity during baseline, three- and six-month follow-up.

| **Variable** | **T0** | **T1** | **T2** |
| --- | --- | --- | --- |
|  | **Mean (95% CI)** | **Mean (95% CI)** | **Mean (95% CI)** |
| **Global quality of life** | 69.1 (64.7 to 73.4) | 69.2 (63.8 to 74.6) | 71.9 (67.1 to 76.9) |
| **Functional scales** |  |  |  |
| Physical functioning | 88.3 (85.9 to 90.7) | 71.8 (65.3 to 78.2) | 68.5 (61.7 to 75.2) |
| Emotional functioning | 81.3 (76.7 to 85.8) | 79.2 (75.2 to 83.3) | 76.9 (73.1 to 80.9) |
| **Symptom scales** |  |  |  |
| Fatigue | 25.9 (19.9 to 31.9) | 22.1 (17.4 to 26.7) | 24.5 (19.8 to 29.3) |
| Nausea and vomiting | 10.6 (6.6 to 14.6) | 14.6 (7.6 to 21.5) | 6.6 (3.3 to 10.0) |
| Pain | 28.0 (22.2 to 33.8) | 24.1 (19.4 to 28.8) | 29.6 (24.3 to 34.8) |
| Dyspnea | 3.3 (-0.5 to 7.0) | 7.8 (3.1 to 12.5) | 7.8 (3.4 to 12.3) |
| Insomnia | 21.9 (15.1 to 28.8) | 12.1 (7.5 to 16.7) | 8.5 (5.8 to 11.3) |
| Appetite loss | 7.6 (2.6 to 12.7) | 11.0 (7.2 to 14.9) | 9.5 (6.5 to 12.5) |
| Constipation | 5.5 (1.9 to 9.1) | 4.4 (2.5 to 6.3) | 4.8 (3.0 to 6.5) |
| **Visual analogue scale** | 3.3 (2.5 to 4.0) | 2.6 (1.9 to 3.3) | 2.8 (2.1 to 3.5) |

The model is adjusted for age, place of residence, marital status and KPS score at baseline.

*T0* baseline, *T1* three-month follow-up, *T2* six-month follow-up, *CI* confidence interval.
